# Effect of *MBOAT7* variant on hepatitis B and C infections in Moroccan patients

**DOI:** 10.1038/s41598-018-30824-9

**Published:** 2018-08-16

**Authors:** Sayeh Ezzikouri, Raouia Elfihry, Hajar Chihab, Mohcine Elmessaoudi-Idrissi, Imane Zaidane, Fatima Zahra Jadid, Adnane Karami, Mohamed Tahiri, Abdellah Elhabazi, Mostafa Kabine, Mohammed Chair, Pascal Pineau, Soumaya Benjelloun

**Affiliations:** 1 Virology Unit, Viral Hepatitis Laboratory, Institut Pasteur du Maroc, Casablanca, Morocco; 2 Santé et Environnement, Faculté des Sciences Aïn Chock, Casablanca, Morocco; 3 Laboratoire de Biotechnologie, Biochimie et Nutrition, Université Chouaib Doukkali, Faculté des Sciences d’El Jadida, El Jadida, Morocco; 4 Service d’Hépato-Gastro-Entérologie, CHU Ibn Rochd, Casablanca, Morocco; 5 Unité “Organisation Nucléaire et Oncogenèse”, INSERM U993, Institut Pasteur, Paris, France

## Abstract

The outcomes of HBV and HCV infections are associated both with viral and host genetic factors. Here, we explore the role of a genetic variation located in membrane-bound O-acyltransferase domain-containing protein 7 (*MBOAT7)* gene on spontaneous clearance of HBV and HCV infections and on liver fibrosis. We genotyped *MBOAT7* rs641738 polymorphism in 971 consecutive Moroccan subjects, including 288 patients with chronic hepatitis C (CHC), 98 cases with spontaneous clearance of HCV, 268 patients with chronic hepatitis B (CHB), 126 spontaneously cleared HBV infections and 191 healthy controls. *MBOAT7* rs641738 variant is not associated with spontaneous clearance of HBV (OR = 0.67, 95% CI: 0.39–1.14; p = 0.131) and HCV infections (OR = 1.33, 95% CI: 0.79–2.23; p = 0.278). Furthermore, multivariable logistic regression analysis adjusted for biologically relevant covariates and potential confounders associated with the risk of liver disease progression revealed that *MBOAT7* rs641738 is not associated either with fibrosis progression in CHC group (OR = 1.12; 95% CI: 0.55–2.28; p = 0.761) or with chronic progressive state in CHB patients (OR = 0.81; 95% CI: 0.41–1.61; p = 0.547). We conclude that the variant *MBOAT7* rs641738 genotype is not associated with spontaneous clearance of HBV and HCV infections or with the progression of liver disease in chronic hepatitis B or C in a genetic context of Mediterranean patients.

## Introduction

Hepatitis B virus (HBV) and hepatitis C virus (HCV) infections are major causes of acute and chronic liver disease, resulting in an estimated 1.4 million deaths annually^1,2^. Worldwide, it is estimated that 248 million people are living with chronic HBV infection (CHB), and that 110 million persons are HCV-antibody positive among whom 80 million suffer from a *bona fide* chronic infection (CHC). Thus, the burden of persistent infections with HBV or HCV remains disproportionately high in low- and middle-income countries, particularly in Asia and Africa^1^. Persons with chronic HBV or HCV infections are at high risk to develop progressive hepatic fibrosis and subsequent cirrhosis, two conditions associated with a higher risk of hepatocellular carcinoma (HCC). A recent genome-wide association study in individuals of European descent (with subsequent validation in two independent European cohorts) identified a novel single-nucleotide polymorphism (SNP; rs641738) in the membrane bound O-acyltransferase domain containing 7 gene (*MBOAT7*), as associated with alcoholic cirrhosis^3^. Furthermore, the rs641738 *MBOAT7* SNP was subsequently found to influence histological liver damage in nonalcoholic fatty liver disease (NAFLD), hepatitis C and hepatitis B^4–10^. Interestingly, previous studies have shown that the rs641738 T allele is associated with lower MBOAT7 mRNA levels and protein expression, as well as reduced arachidonoyl-phosphatidylinositol/total phosphatidylinositol ratios coupled with higher values of oleyl- or linoleyl-phosphatidylinositol/total phosphatidylinositol ratios. These disturbances suggest that the rs641738 T allele may modulate the inflammation process independently of the etiology of the liver disease by down-regulating the *MBOAT7* expression and protein synthesis^4–6^. Despite the wealth of data about the putative role of *MBOAT7* variation in the progression of liver diseases, no data assessing the relationship between the *MBOAT7* rs641738 genetic variant and earlier steps of the disease *ie*. spontaneous clearance of HBV and HCV infection is available so far. In the present study, we assessed whether the *MBOAT7* rs641738 polymorphism is affecting the resolution of HBV and HCV infections as well as the progression of liver disease in a well-characterized Moroccan cohort of patients with chronic infections.

## Materials and Methods

### Patients and healthy controls

**Figure 1:**
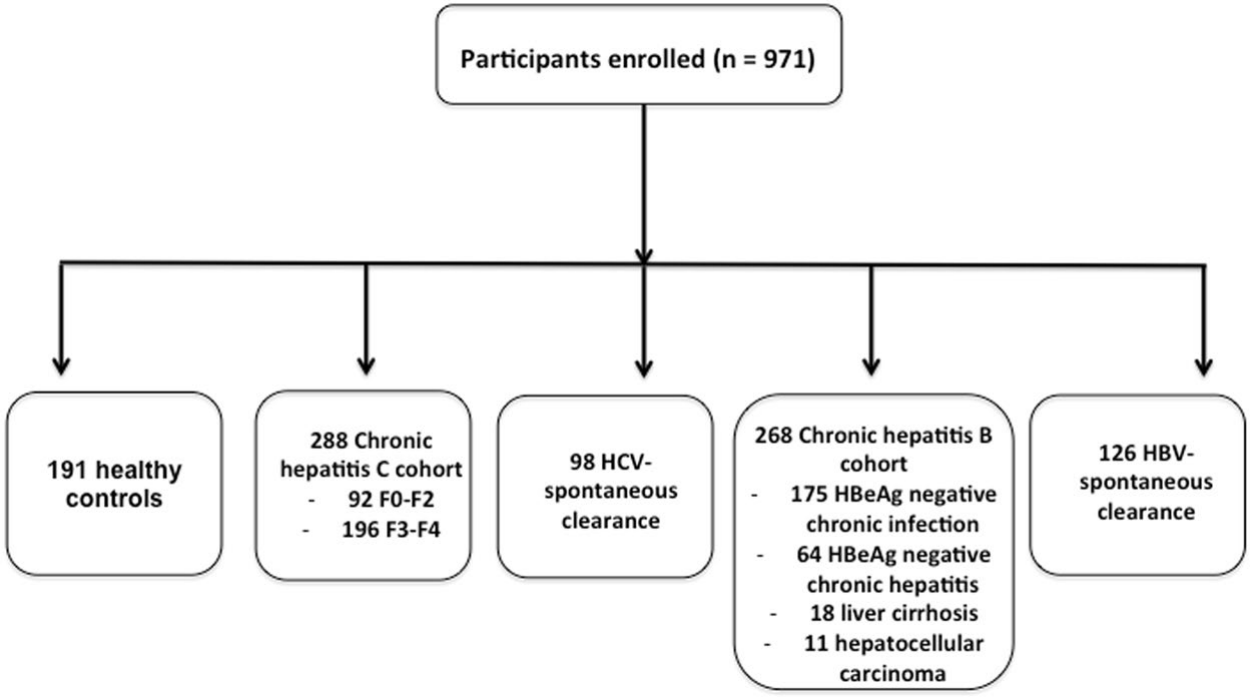
Study profile.

This study prospectively included 971 consecutive Moroccan subjects (Fig. 1), including 288 with CHC, 98 formerly HCV-infected patients who underwent spontaneous clearance, 268 with CHB, 126 subjects who spontaneously cleared HBV (seropositive for anti-HBs and anti-HBc) and 191 healthy controls. The subjects were enrolled at the Medical Center of Biology at the Pasteur Institute of Morocco and Service of Medicine B CHU Ibn Rochd hospital, Casablanca from June 2004 to January 2015. CHC are defined as patients persistently positive for anti-hepatitis C virus (anti-HCV) antibodies and HCV RNA by a quantitative reverse transcriptase-polymerase chain reaction (qRT-PCR) for at least six months. In these patients, histological features were assessed noninvasively using FibroTest-ActiTest combining α-2-macroglobulin, GGT, apolipoprotein A1, haptoglobin, total bilirubin, age and gender (Biopredictive, Paris, France). The CHC patients were stratified according to; fibrosis as absent/mild (F0–F2, n = 92) or significant (F3-F4, n = 196). The group with HCV-spontaneous clearance was positive for anti-HCV and negative for HCV RNA by qRT-PCR according to least two measurements more than 6 months apart.

The diagnosis of CHB was based on HBsAg-seropositivity for more than six months. The CHB patients were classified, according to clinical practice guidelines on the management of hepatitis B virus infection^11^, into HBeAg negative chronic infection group (n = 175) with persistently normal serum alanine aminotransferase (ALT) level and low HBV-DNA level (<3.3 log_10_ IU/mL) during at least 6 months of follow up, HBeAg negative chronic hepatitis patients (n = 64) defined by persistent or intermittent elevation in ALT level and HBV-DNA level (>3.3 log_10_ IU/mL) for at least 6 months and histological signs of moderate/severe necro-inflammation and patients with liver cirrhosis (LC, n = 18) and HCC (n = 11). LC was diagnosed by ultrasonography and clinical criteria indicating portal hypertension (ascites, esophageal varices, etc). The diagnosis of HCC was based on serum alpha-foetoprotein levels (AFP), imaging showing the characteristic features of HCC and/or when possible histological assessment of tissues samples. The HBeAg negative chronic hepatitis, LC and HCC (n = 93) patients were considered to have chronic progressive liver disease, and were gathered in a chronic hepatitis B group when compared with HBeAg negative chronic infection patients group. Subjects with spontaneous clearance from HBV infection were positive for antibody against hepatitis B core antigen and anti-HBs.

Healthy controls were negative for hepatitis serological markers and with normal serum levels of ALT and aspartate aminotransferase (AST) with no current or past history of liver disease. Patients with evidence of co-infection with human immunodeficiency virus, presence of autoimmune liver disease were excluded.

### Serological and molecular analyses

Sera were tested for HBsAg, anti-HCV (Axsym/Architect, Abbott Diagnostics, Wiesbaden-Delkenheim, Germany) and anti-HIV (Genscreen Ag/Ab HIV Ultra, Biorad, Marnes La Coquette, France). Plasma HCV-RNA was measured by qPCR using COBAS AmpliPrep/COBAS TaqMan (Roche Diagnostics, Germany). HCV RNA level below the detection threshold (50IU/mL) were scored as negative for HCV RNA. ALT, AST, gamma-glutamyl transpeptidase (GGT), bilirubin, total cholesterol, high density lipoprotein (HDL), low density lipoprotein (LDL) and triglycerides were collected at time of blood sampling by two separate interviews. The study was conformed to the ethical guidelines of the 1975 Declaration of Helsinki and approved by the ethics committee of the Faculty of Medicine of Casablanca. All participants of this study gave their informed consent.

### DNA isolation and *MBOAT7* rs641738 Genotyping

Genomic DNA was isolated from the peripheral blood mononuclear cells as described previously^12,13^. Genotyping for *MBOAT7* rs641738 was undertaken using the TaqMan SNP genotyping allelic discrimination method (Applied Biosystems, Foster City, CA, USA). All genotyping was blinded to clinical variables and SNP determination was scored by two independent investigators (R.E and H.C) in order to ensure correct typing results.

### Statistical analysis

For descriptive statistics, continuous variables are shown as median and range. Categorical variables are presented as number and proportion. Hardy-Weinberg equilibrium was performed by a χ^2^ test with 1 degree of freedom. Comparisons of continuous variables between groups were conducted using ANOVA. We examined five potential genetic models that might explain the effect of *MBOAT7* rs641738 on spontaneous clearance outcomes: co-dominant; dominant; recessive; over dominant; and additive. We investigated which model was the most appropriate by calculating the Akaike information criterion (AIC) values^14^. The lowest AIC is indicative of the best fit. Multivariable logistic regression analysis were adjusted for biologically relevant covariates and potential confounders associated with the risk of liver disease progression in chronic hepatitis C patients (age, sex, viral load, ALT, serum cholesterol, HDL, LDL, triglycerides, GGT and *MBOAT7* rs641738 genotype) and (age, sex, viral load, ALT, AST and *MBOAT7* rs641738 genotype) in CHB patients. All statistical procedures were performed with R software for Windows and the effect of genetic polymorphism on spontaneous clearance was examined with the SNPassoc R package (https://www.r-project.org). P values less than 0.05 were considered statistically significant. All statistical tests were two-sided.

## Results

### Clinical and biochemical characteristics of the patients

The demographic data, biochemical features, viral load, and clinical features of the studied groups are summarized in Tables 1 and 2.

**Table 1:** Baseline characteristics of healthy subjects, chronic HCV patients and HCV-spontaneous clearance group.

	Healthy control (n = 191)	Chronic HCV infection (n = 288)	HCV-Spontaneous clearance (n = 98)
Age, years	56 [18–93]	63 [20–88]	58[20–76]
Gender, (%)			
Male	63 (32.98)	104 (36.11)	40 (40.82)
Female	128 (60.02)	184 (63.89)	58 (59.18)
Alanine aminotransferase, (IU/L)	30 [12–54]	63 [12–361]	28 [12–92]
Aspartate aminotransferase, (IU/L)	25 [12–34]	54 [16–280]	29.50 [14–65]
Gamma-glutamyltransferase, (IU/L)	NA	73 [40–500]	20 [14–65]
Total cholesterol, (g/L)	1.91 [1.09–2.99]	1.50 [0.84–2.87]	1.10 [0.41–6.95]
HDL cholesterol, (g/L)	0.50 [0.24–4.36]	0.51 [0.14–1.27]	0.79 [0.47–1.53]
LDL cholesterol, (g/L)	1.10 [0.4–1.97]	0.82 [0.3–5.11]	3.15 [1.34–5.43]
Triglycerides, (g/L)	1.11 [0.31–4.31]	0.98 [0.48–2.13]	0.46 [0.35–0.69]
Viral load, Log10 (IU/mL)	NA	6.03 [1.78–7.50]	—
Viral genotypes (%)			
1/2	—	64.5/35.5	—
*MBOAT7* rs641738 variant, (%)			
CC	54 (28.27)	90 (31.25)	25 (25.51)
CT	107 (56.02)	144 (50)	51 (52.04)
TT	30 (15.71)	54 (18.75)	22 (22.45)
C allele	0.563 ± 0.024	0.562 ± 0.020	0.515 ± 0.035
T allele	0.437 ± 0.024	0.438 ± 0.020	0.485 ± 0.035

NA: data not available.

**Table 2:** Baseline characteristics of healthy subjects, chronic HBV patients and HBV-spontaneous clearance group.

	Chronic HBV infection (n = 268)	HBV-Spontaneous clearance (n = 126)
Age, years	42 [19–78]	55 [19–85]
Gender, (%)		
Male	102 (38.35)	71 (56.35)
Female	164 (61.65)	55 (43.65)
Alanine aminotransferase, (IU/L)	45 [9–400]	31 [13–106]
Aspartate aminotransferase, (IU/L)	35 [10–245]	26 [15–126]
Viral load, Log10 (IU/mL)	3.37 [1.08–9]	NA
Viral genotypes (%)		
D/A	91/9	NA
*MBOAT7* rs641738 variant, (%)	80 (30.07)	41 (32.54)
CC	119 (44.74)	62 (49.21)
CT	67 (25.19)	23 (18.25)
TT		
C allele	0.524 ± 0.023	0.571 ± 0.031
T allele	0.476 ± 0.023	0.429 ± 0.031

### *MBOAT7* rs641738 minor allele frequency in Moroccan healthy subjects

To estimate the frequencies of the *MBOAT7* rs641738 genotypes in Morocco population, the rs641738 SNP was genotyped in 191 healthy controls. The distribution of *MBOAT7* rs641738 genotypes for healthy control group complied with Hardy-Weinberg equilibrium (p = 0.077). Overall, the genotype distribution at the rs641738-C/T loci was identified as follows: CC homozygous in 54 (28.27%) individuals, heterozygosity in 107 (56.02%) and homozygote TT in 30 (15.71%). The minor allele (T) frequency (MAF) was 0.437 in Moroccan population (Table 1).

### Association between the *MBOAT7* rs641738 polymorphism and spontaneous clearance or disease progression of HBV infection

First, we analyzed the effect of the rs641738 SNP on HBV infection outcomes. The recessive model for the minor allele (T) best fitted the data and was the most appropriate, as it had the lowest Akaike Information Criterion (AIC) value. The distribution of *MBOAT7* rs641738 genotypes did not differ with the status of HBV-infection (Supplementary Table 1).

Next, we evaluated whether the functional impact of *MBOAT7* rs641738 polymorphism was related to disease progression in HBV infection by comparing the genotype frequencies between the HBV chronic infection patients and chronic hepatitis B group (HBeAg negative chronic hepatitis, LC and HCC). No significant differences were detected between the two groups regarding *MBOAT7* rs641738 polymorphism. However, a significant association was found male sex, age, ALT, AST and viral load were associated with risk of progressive liver disease (Table 3).

**Table 3:** Factors associated with progression of liver disease in chronic hepatitis B patients.

	HBV chronic infection group (n = 175)	Chronic hepatitis B group (n = 93)	OR	95% CI	P value
Age, years	42 [33.2–50.8]	41 [34.0–51.5]	1	0.98–1.02	0.827
Male sex	95 (55.2%)	66 (71.7%)	1.15	0.99–1.84	0.013
ALT, (IU/L)	45 [33.0–45.0]	80 [62.0–92.0]	0.74	0.64–0.86	<0.001
AST, (IU/L)	35 [31.0–35.0]	70 [48.0–105]	0.84	0.79–0.91	<0.001
Viral Load log10 (IU/mL)	2.90 [2.47–3.30]	4.65 [3.86–6.11]	1	1.00–1.00	<0.001
HBV genotype D	164 (93.7%)	84 (90.3%)	1.55	0.50–5.36	0.615
*MBOAT7* rs641738 TT genotype	42 (24%)	25 (26.7%)	0.81	0.41–1.61	0.547

ALT: Alanine aminotransferase; AST: Aspartate aminotransferase; MBOAT7: membrane bound O-acyltransferase domain containing 7 gene; OR: Odds ratio; CI: confidence interval.

### Association between the *MBOAT7* rs641738 polymorphism and spontaneous clearance or disease progression of HCV infection

Regarding HCV infection, the dominant model for the minor allele (T) best fitted the data. The distribution of *MBOAT7* rs641738 genotypes did not differ between HCV-persistent and HCV-cleared groups (Supplementary Table 2).

Next, we tested subsequently the association of rs641738 with fibrosis stage by multiple logistic regression analysis adjusted for age, gender, and viral load. CHC patients were stratified according to fibrosis as absent/mild (F0-F2) or significant (F3-F4). The result indicated that age, male sex, cholesterol, GGT, ALT and AST were associated with advanced fibrosis (p < 0.05). On the other hand, we observed an absence of impact of rs641738 TT genotype on fibrosis progression (OR = 1.12; 95% CI: 0.55–2.28; p = 0.761; Table 4).

**Table 4:** Factors associated with liver fibrosis F3-F4 in chronic hepatitis C patients.

	F0–F2 group (n = 92)	F3-F4 group (n = 196)	OR	95% CI	p-value
Age, years	58.5 [51–67]	65 [58–73]	1.06	1.04–1.09	<0.001
Male sex (%)	23 (25.0%)	81 (41.3%)	2.1	1.23–3.71	0.011
Serum cholesterol, (g/L)	1.64 [1.36–1.85]	1.42 [1.25–1.67]	0.23	0.07–0.75	0.013
HDL, (g/L)	0.52 [0.42–0.66]	0.47 [0.37–0.60]	0.14	0.01–1.73	0.213
LDL, (g/L)	0.89 [0.60–1.15]	0.80 [0.50–0.92]	0.42	0.10–1.68	0.163
Triglycerides, (g/L)	0.96 [0.81–1.27]	0.98 [0.78–1.21]	1.12	0.41–3.06	0.805
Gamma-glutamyltransferase, (IU/L)	27.0 [19.0–40.0]	63.0 [37.5–114]	1.02	1.01–1.03	<0.001
ALT, (IU/L)	40.5 [28.2–61.8]	80 [52.5–122]	1.02	1.01–1.04	<0.001
AST, (IU/L)	33.5 [27.0–44.2]	71.0 [54.0–117]	1.04	1.02–1.06	<0.001
*MBOAT7* rs641738 TT genotype	19 (20.7%)	35 (17.9%)	1.12	0.55–2.28	0.761

HDL: High density lipoprotein; LDL: low density lipoprotein; ALT: Alanine aminotransferase; AST: Aspartate aminotransferase; MBOAT7: membrane bound O-acyltransferase domain containing 7 gene; OR: Odds ratio; CI: confidence interval.

### Biochemical and metabolic characteristics according to rs641738 genotype

**Figure 2:**
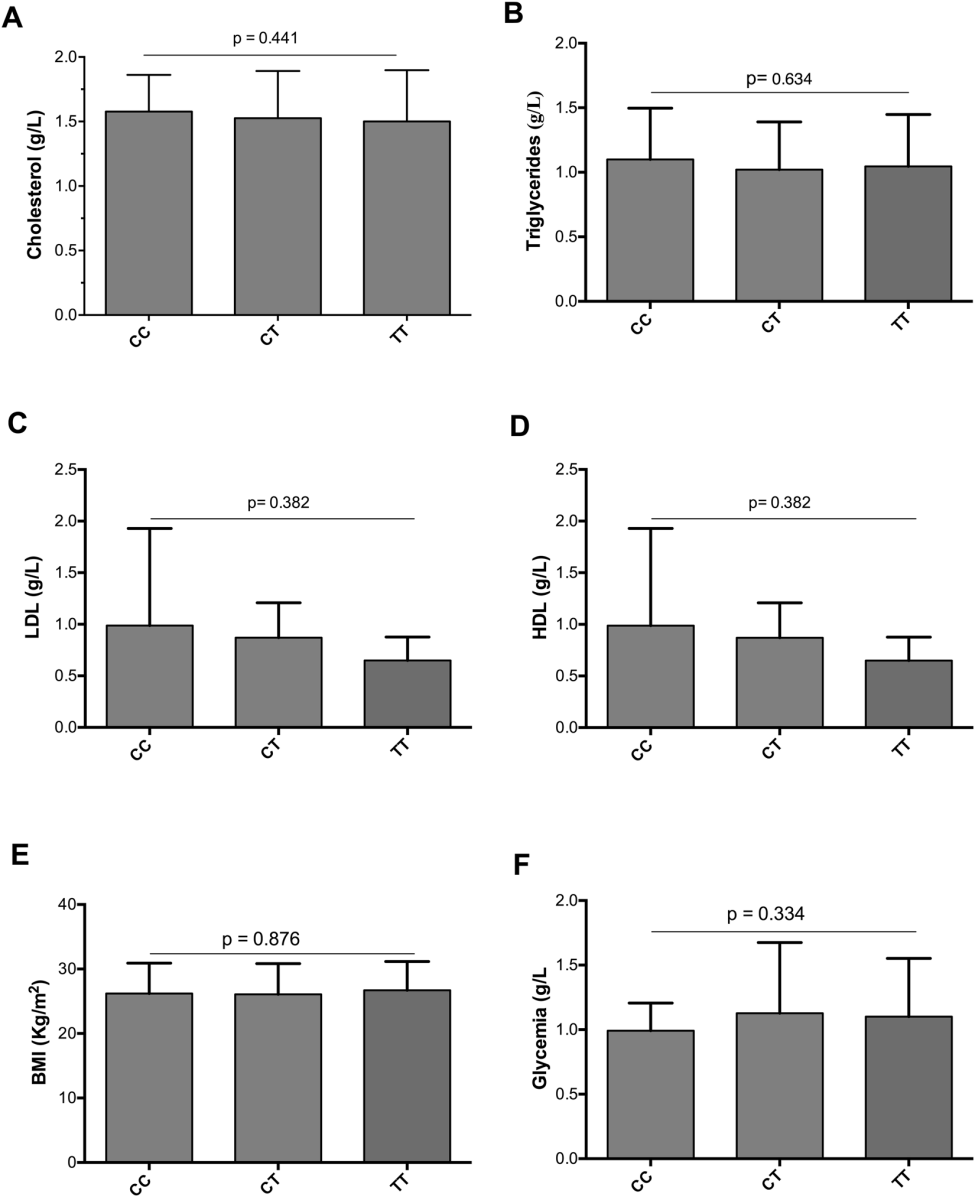
Association of rs641738 genotype with lipid profile and blood glucose in CHC patients. (**A**) Summary of the differences of serum cholesterol according to genotypes of *MBOAT7* rs641738. (**B**) Triglycerides. (**C**) LDL. (**D**) HDL. (**E**) BMI. (**F**) Glycemia. Data are expressed as the mean and standard deviation. Statistical analyses were performed using ANOVA.

**Figure 3:**
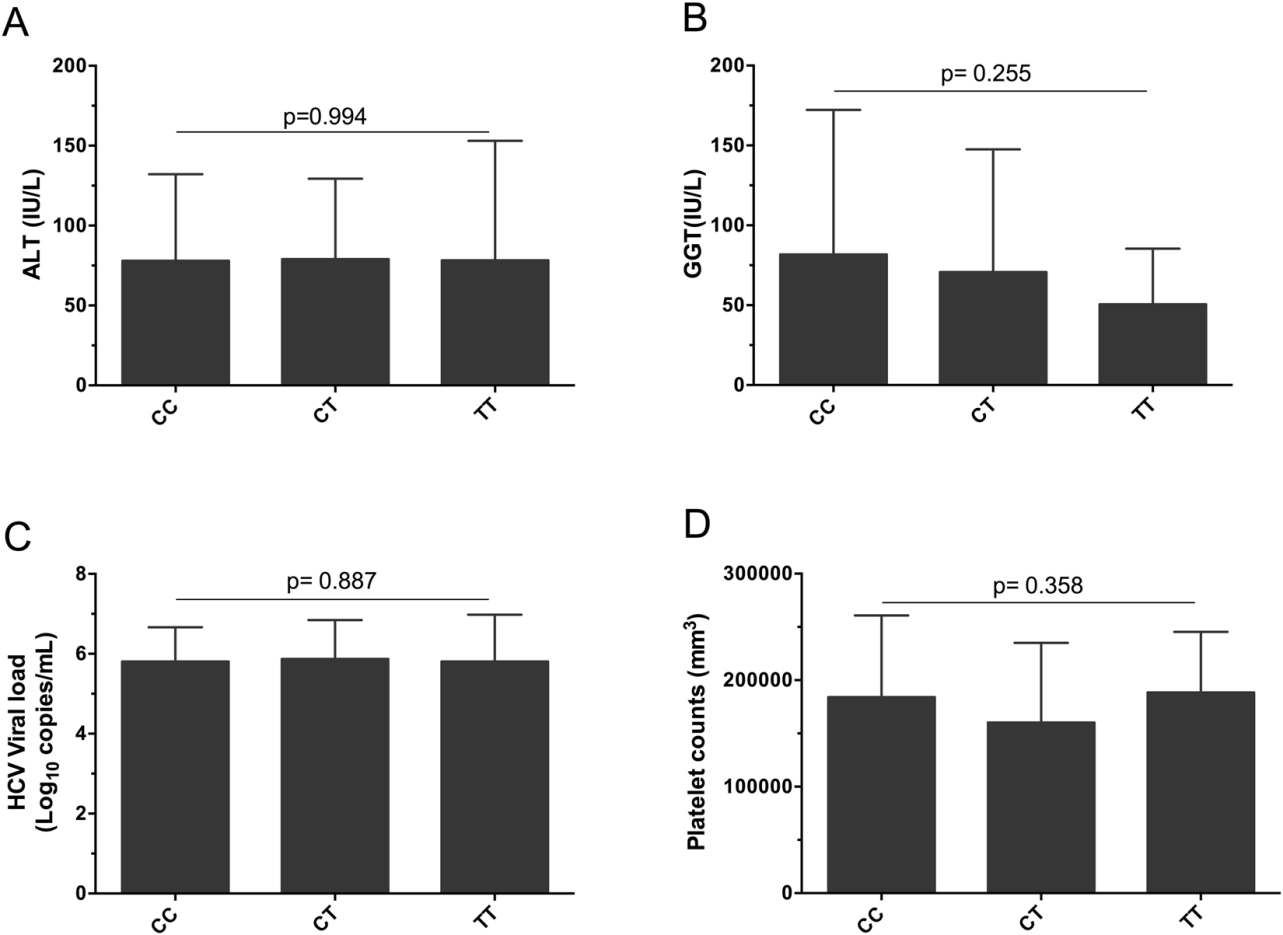
Association of rs641738 genotype with liver injury in CHC group. (**A**) Comparison between CC, CT and TT in terms of ALT. (**B**) GGT. (**C**) HCV viral load. (**D**) Platelet counts. Data are expressed as the mean and standard deviation. Statistical analyses were performed using ANOVA.

No significant associations were found with any available biochemical features such as liver enzymes (Fig. 2), platelet counts, lipid profile, anthropometric traits or HCV-RNA according to rs641738 genotype in CHC group (Fig. 3). Furthermore, no significant results were found for the features analyzed in CHB patients (Supplementary Fig. 1).

## Discussion

CHB and CHC are characterized by liver inflammation that promotes, on the long term, fibrosis, cirrhosis and HCC^15,16^. It is known that genetic variation of the host, viral and environmental factors are modulating disease progression^17,18^. In the present study, we investigated the role of rs641738 C > T genetic variant in *MBOAT7* in Moroccan patients with a broad spectrum of liver disease. In Moroccan population, the MAF frequency was 0.44 which is in line with the frequencies reported in Caucasians population but lower than those reported Asian populations^6,8^. To our knowledge, this study is the first description of the relationship between *MBOAT7* rs641738 polymorphism and clearance of HBV and HCV. The major finding of the present study was the lack of association of rs641738 C > T genetic variant in the *MBOAT7* gene and spontaneous resolution of HBV and HCV infections.

The *MBOAT7* rs641738 polymorphism was first identified as a predisposing locus for cirrhosis in alcoholic liver disease^3^. In the setting of CHB and CHC, the immune system initially attempts to eradicate the HBV and HCV, while it probably promotes progressive liver damage and fibrosis in the same time. In the present study, we found that *MBOAT7* rs641738 is not associated with progression in both CHB and CHC. Our data are, thus, at odd with previous studies showing that *MBOAT7* rs641738 variant accelerates the progression to fibrosis in CHC and CHB of Caucasian patients^6,8^. In addition, the *MBOAT7* rs641738 SNP variant was shown to predispose Caucasian subjects to liver fibrosis subjects with excessive alcohol intake^3^ and to progression of nonalcoholic fatty liver disease (NAFLD)^4,5^. In contrast, a Korean study did not reveal any association between *MBOAT7* rs641738 SNP and the histologic severity of NAFLD^19^. The discrepancy between results found in studies conducted on subjects with different anthropological background may be related to allele frequency differences between populations, or to the genetic architecture of the populations studied and its inherent set of epistasis^4,8^. We speculate that the effect of *MOBAT7* rs641738 variant on the development of liver disease progression may be influenced by ethnicity^4,8,10^. Moreover, concerning HBV infection, we compared patients with HBV infection without hepatitis versus patients with infection and hepatitis. This could explain at least in part the different findings of this study compared to previous study^8^. Nowadays, the functional mechanisms of MBOAT7 action in liver diseases remain unknown. MBOAT transfers arachidonic acid to lysophosphatidylinositol to produce arachidonic acid–containing phosphatidylinositol^20^. Moreover, previous study showed that MBOAT7 is independently associated with inflammation, oxidative stress, macrophage activation and the transition to early fibrosis^6^.

Our data did not reveal any significant associations with clinical characteristics such as liver enzymes, lipid profile, HCV-RNA, or HBV-DNA. This result seems to be in line with previous findings^6,8,10^. However, a higher ALT levels is observed in individuals carrying the T allele^8,21^.

As a conclusion, the *MBOAT7* rs641738 variant is not associated with spontaneous resolution of HBV and HCV infections. Furthermore, our data show that *MBOAT7* rs641738 polymorphism does not predispose infected patients to progress to an advanced stage. Our study has some limitations, including missing data on the MBOAT expression in HCV/HBV-infected liver biopsies. Moreover, we are fully aware that the limitation of the current study is the relatively small size of the cohort examined. Further surveys warrant the recruitment of a larger cohort and future analyses of additional polymorphisms in *MBOAT7* gene and Transmembrane 6 superfamily 2 *(TM6SF2)* E167K variant will help to clarify their role in HCV/HBV infections and progression. Overall, further studies elucidating the mechanistic function and on large and others cohorts, notably North African patients with NAFLD, are warranted to definitively refute or confirm an effect of *MOBAT7* rs641738 variant on liver diseases progression.

## Electronic supplementary material

Supplementary Information
