# Supplementary material for: Effect of MBOAT7 variant on hepatitis B and C infections in Moroccan patients
Source: Sci Rep. 2018 Aug 16;8:12247. doi: 10.1038/s41598-018-30824-9 (PMC6095921; doi:10.1038/s41598-018-30824-9)

Supplementary Information

Effect of *MBOAT7* variant on hepatitis B and C infections in Moroccan patients

Sayeh Ezzikouri [1*], Raouia Elfihry [1,2#], Hajar Chihab [1,3#], Mohcine Elmessaoudi-Idrissi [1], Imane Zaidane [1], Fatima Zahra Jadid [1], Adnane Karami [1], Mohamed Tahiri [4], Abdellah Elhabazi [3], Mostafa Kabine [2], Mohammed Chair [3], Pascal Pineau [5], Soumaya Benjelloun [1]

[1]Virology Unit, Viral Hepatitis Laboratory, Institut Pasteur du Maroc. Casablanca, Morocco

[2]Santé et Environnement, Faculté des Sciences Aïn Chock, Casablanca, Morocco

[3]Laboratoire de Biotechnologie, Biochimie et Nutrition, Université Chouaib Doukkali, Faculté des Sciences d'El Jadida, El Jadida, Morocco

[4]Service d'Hépato-Gastro-Entérologie, CHU Ibn Rochd, Casablanca, Morocco

[5]Unité "Organisation Nucléaire et Oncogenèse", INSERM U993, Institut Pasteur, Paris, France

[#] These authors contributed equally to the manuscript

[*]Correspondence should be addressed to Dr. Sayeh Ezzikouri, Virology Unit, Viral Hepatitis Laboratory, Institut Pasteur du Maroc, 1 Place Louis Pasteur, 20360 Casablanca-Morocco. Tel: +212 5 22434470; Fax: +212522260957; E.mail: sayeh.ezzikouri@pasteur.ma

Supplementary table 1: Effect of *MBOAT7* rs641738 polymorphism on the oucomes HBV infection

	**Chronic HBV infection (N= 266)**	**%**	**HBV-Spontaneous clearance (N= 126)**	**%**	**OR**	**lower**	**upper**	**p-value**	**AIC***
Codominant									
C/C	80	30.1	41	32.8	1			0.320452725	493.8
C/T	119	44.7	61	48.8	1	0.61	1.63		
T/T	67	25.2	23	18.4	0.67	0.37	1.23		
Dominant									
C/C	80	30.1	41	32.8	1			0.587778305	493.7
C/T-T/T	186	69.9	84	67.2	0.88	0.56	1.39		
Recessive									
C/C-C/T	199	74.8	102	81.6	1			0.131386933	491.8
T/T	67	25.2	23	18.4	0.67	0.39	1.14		
Overdominant									
C/C-T/T	147	55.3	64	51.2	1			0.452468547	493.5
C/T	119	44.7	61	48.8	1.18	0.77	1.8		
log-Additive									
0.1.2	266	68	125	32	0.84	0.62	1.12	0.228912846	492.6

*Akaike information criterion

Supplementary table 2: Effect of *MBOAT7* rs641738 polymorphism on the oucomes of HCV infection

	**HCV-Spontaneous clearance (N= 98)**	**%**	**Chronic HCV infection (N= 288)**	**%**	**OR**	**lower**	**upper**	**p-value**	**AIC***
Codominant									
C/C	90	31.2	25	25.5	1			0.499068595	442
C/T	144	50	51	52	1.28	0.74	2.2		
T/T	54	18.8	22	22.4	1.47	0.75	2.85		
Dominant									
C/C	90	31.2	25	25.5	1			0.278345397	440.2
C/T-T/T	198	68.8	73	74.5	1.33	0.79	2.23		
Recessive									
C/C-C/T	234	81.2	76	77.6	1			0.431348678	440.8
T/T	54	18.8	22	22.4	1.25	0.72	2.19		
Overdominant									
C/C-T/T	144	50	47	48	1			0.727026774	441.3
C/T	144	50	51	52	1.09	0.69	1.72		
log-Additive									
0.1.2	288	74.6	98	25.4	1.21	0.87	1.69	0.246653287	440

*Akaike information criterion

Legends to Supplementary Figures

Figure S1: Association of rs641738 genotype with liver injury in CHB group. (A) Comparison between CC, CT and TT in terms of ALT. (B) AST. (C) HBV viral load. Data are expressed as the mean and standard deviation. Statistical analyses were performed using ANOVA.

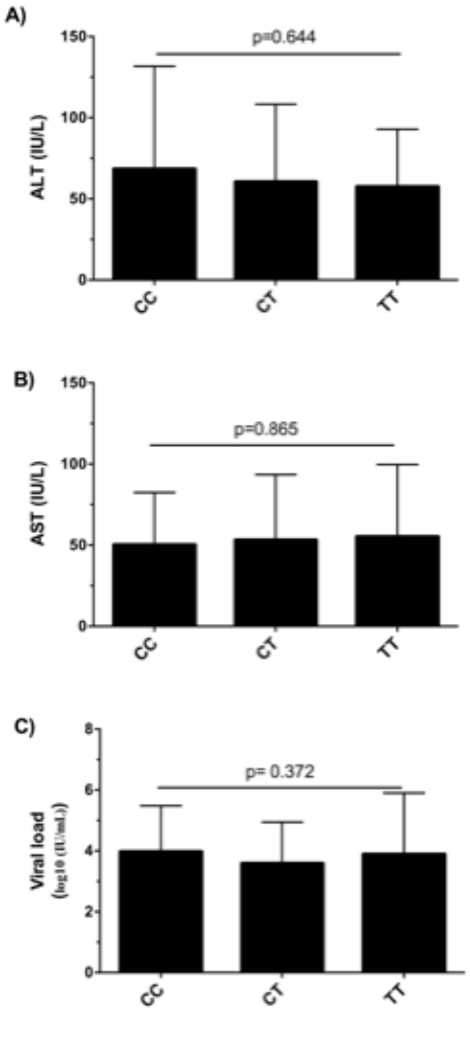

Supplement: Supplementary file 1 — Supplementary Information [file 41598_2018_30824_MOESM1_ESM.pdf]
